# Clinical application of circulating tumor cells in breast cancer: overview of the current interventional trials

**DOI:** 10.1007/s10555-012-9398-0

**Published:** 2012-11-06

**Authors:** François-Clément Bidard, Tanja Fehm, Michail Ignatiadis, Jeffrey B. Smerage, Catherine Alix-Panabières, Wolfgang Janni, Carlo Messina, Costanza Paoletti, Volkmar Müller, Daniel F. Hayes, Martine Piccart, Jean-Yves Pierga

**Affiliations:** 1 Department of Medical Oncology, Institut Curie, 26 rue d’Ulm, 75005 Paris, France; 2 Université Paris Descartes, Paris, France; 3 Department of Gynecology and Obstetrics, University of Tübingen, Tübingen, Germany; 4 Department of Medical Oncology, Institut Jules Bordet, Brussels, Belgium; 5 Breast Oncology Program, University of Michigan Comprehensive Cancer Center, Ann Arbor, MI USA; 6 Laboratory of Rare Human Circulating Cells, University Medical Center, UM1, Montpellier, France; 7 Department of Gynecology and Obstetrics, University of Düsseldorf, Düsseldorf, Germany; 8 European Organization for Research and Treatment of Cancer (EORTC) Headquarters, Brussels, Belgium; 9 Department of Gynecology, University Medical Center Hamburg-Eppendorf, Hamburg, Germany

**Keywords:** Breast cancer, CellSearch®, Circulating tumor cells

## Abstract

In 2004, circulating tumor cells (CTC) enumeration by the CellSearch® technique at baseline and during treatment was reported to be associated with prognosis in metastatic breast cancer patients. In 2008, the first evidence of the impact of CTC detection by this technique on survival of cM0(i+) patients were reported. These findings were confirmed by other non-interventional studies, whereas CTC were also investigated as a surrogate for tumor biology, mainly for HER2 expression/amplification. The aim of this report is to present the current prospective large interventional studies that have been specifically designed to demonstrate that CTC enumeration/characterization may improve the management of breast cancer patients: STIC CTC METABREAST (France) and Endocrine Therapy Index (USA) assess the CTC-guided hormone therapy *vs* chemotherapy decision in M1 patients; SWOG0500 (USA) and CirCe01 (France) assess the CTC count changes during treatment in metastatic patients; DETECT III (M1 patients, Germany) and Treat CTC (cM0(i+) patients, European Organization for Research and Treatment of Cancer/Breast International Group) assess the use of anti-HER2 treatments in HER2-negative breast cancer patients selected on the basis of CTC detection/characterization. These trials have different designs in various patient populations but are expected to be the pivotal trials for CTC implementation in the routine management of breast cancer patients.

## Introduction

Circulating tumor cells (CTC) designate cancer cells that are detected in the blood of cancer patients. Their detection, quantification, and characterization represent a new window on cancer dissemination and have opened major perspectives for both biological and clinical research on the metastatic process. Commonly accepted endpoints in clinical research for both nonmetastatic and metastatic breast cancer patients are detailed in Box 1. Technically, as most carcinomas do not have specific and recurrent DNA mutation or fusion transcripts, CTC isolation methods rely on the detection of cells that express epithelial-related markers in blood. Since the normal components of human whole blood are mesenchymally derived, they are not identified using this strategy; this principle is similar to the detection of isolated breast tumor cells in axillary lymph node in pN0(i+) breast cancer patients. In 2012, the standard for CTC detection remains the CellSearch® system (Veridex), which is still the only system that has been approved by the FDA for *in vitro* diagnosis purposes. In 2004, a seminal study with this technique showed that CTC count was an independent prognostic factor for both progression-free (PFS) and overall (OS) survival in metastatic (M1) breast cancer patients [1]. In this report, the threshold of ≥5 CTC/7.5 ml to define the poor prognosis group was “learned” from a training group (*n* = 102 patients) and validated in another group of patients (*n* = 75 patients). This prognostic value for PFS and OS has been repeatedly validated in smaller following studies [2–4]. Unsurprisingly, a pooled analysis confirmed these results in multivariate analysis [5]. A prospective study “IC 2006-04,” specifically designed and powered to assess the prognostic value of CTC count changes in patients treated by first-line chemotherapy (with or without targeted therapy), had the same conclusions in multivariate analysis and supported the use of the ≥5 CTC/7.5 ml threshold [6]. Serum markers have been also prospectively assessed [7] and were not prognostic markers in multivariate analysis. The only issue appeared with the use of targeted therapy, namely trastuzumab and bevacizumab, which decrease profoundly the CTC count: some reports suggested that this decrease might impact its prognostic value [8, 9]. Moreover, recent data have suggested an adverse prognostic value of CTC detection in nonmetastatic breast cancer [10–12]. At the same time, HER2 expression on CTCs using the CellSearch® system was studied in the neoadjuvant setting [13] and across all breast cancer stages from preinvasive lesions to overt metastatic disease [14].

Box 1. Clinical settings and endpoints
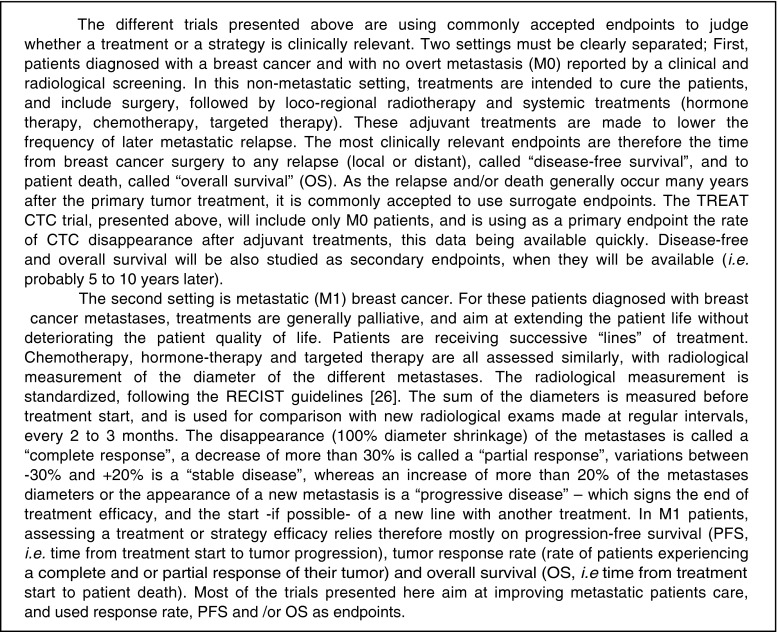


With these numerous non-interventional studies published, interventional controlled “phase III” trials were needed to demonstrate that the use of CTC enumeration and monitoring could improve the outcome of breast cancer patients and/or lower the medical costs paid by the patient or its insurer. The aim of this article is to report and discuss the different interventional trials currently ongoing or on the edge of starting that have been set up by different CTC research groups in the world. Basically, CTCs are investigated as prognostic markers in the STIC CTC METABREAST trial and Endocrine Therapy Index (ETI) study, as early surrogate of chemotherapy efficacy in the SWOG0500 and in the CirCe01 trials, and finally as an indicator of tumor biology in the Treat CTC, DETECT III, and CirCe XXX1 trials.

## STIC CTC METABREAST (France)

### Rationale

As stated above, baseline CTC count has unambiguously demonstrated its very good performance as an independent prognostic marker. Multivariate analyses performed in both the pooled analysis and in the IC 2006–2004 study showed that the other independent prognostic factors were the performance status and hormone receptor (HR) status. Oppositely, the other criteria that are frequently used to choose between hormone therapy and chemotherapy for the treatment of first-line metastatic HR + breast cancer patients (*e.g.*, metastatic sites, metastasis-free interval…) were not independent prognostic factors. It has been then proposed that CTC count may be a better criterion for this important choice than the currently used empiric criteria, which have a low level of evidence (expert consensus).

### Design

**Fig. 1:**
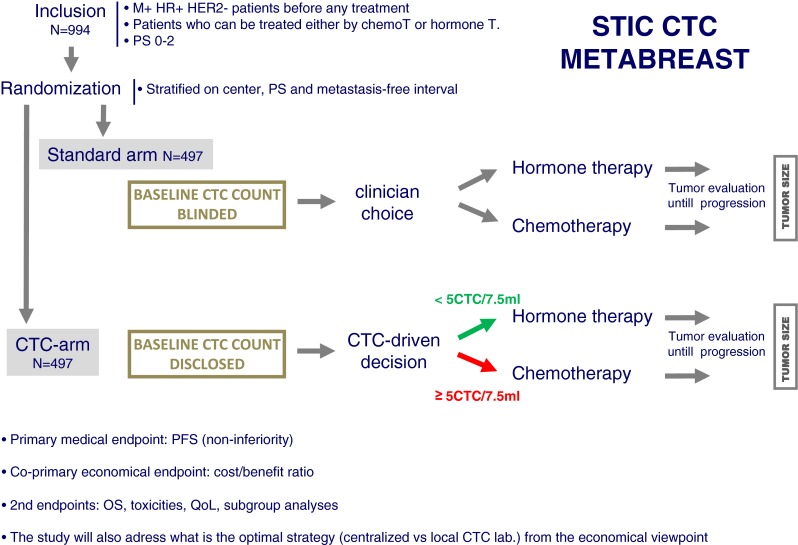
The STIC CTC METABREAST trial design

In the STIC CTC METABREAST trial (NCT01710605), about 1,000 HR + M + breast cancer patients will be randomized between the clinician choice and CTC count-driven choice (Fig. 1). In the CTC arm, patients with ≥5 CTC/7.5 ml will receive chemotherapy whereas patients with <5 CTC/7.5 ml will receive endocrine therapy as first-line treatment. Within each treatment category (hormone or chemotherapy), the treatment type will be the clinician choice, targeted therapy being allowed. The only difference between CTC and standard arms will be the rates of hormone therapy *vs* chemotherapy-based treatment. As every patient will receive a treatment, this pivotal trial has been designed to show a non-inferiority of the CTC arm for PFS (primary clinical endpoint) and a superiority of the CTC arm for the medico-economics study (co-primary endpoint). This trial began in February 2012. Several secondary endpoints are pre-planned (subgroups analyses).

### Funding

The STIC CTC METABREAST trial has been funded by the French Ministry of Health (STIC program, #50 %) and Veridex (#50 %). The promoter is the Institut Curie (Paris).

### Alternate proposal

Based on the same rationale (standard criteria for treatment decision between hormone therapy or chemotherapy may be weaker than CTC count), another strategy is currently developed that takes into account not only the number but also the biology of CTC [15, 16]. Four biological markers are assessed on isolated CTC by immunocytofluorescence in the CellSearch® system: estrogen receptor, Bcl2, HER2, and Ki67. The global level of expression per isolated CTC of each marker is determined, and a score called “Endocrine Therapy Index” is assigned for each marker for that blood draw, based on both the staining intensity and the proportion of positive CTC. The relative weights of the criteria (the number of CTC and the expression of each of the four biomarkers) are used to derive an “ETI score.” An American pilot study to establish the analytical validity of this assay is near completion and a larger trial to test the clinical validity is now being planned.

## SWOG 0500 (USA)

### Rationale

As stated above, several observational studies support the fact that CTC changes during the weeks following the first cycle of chemotherapy is associated with PFS and OS [1]. This observation is intriguing, since other soluble protein markers often rise before they decline—the so-called tumor marker spike. This phenomenon makes assessment of protein biomarkers much less accurate in the early phases of a new treatment, whereas CTC spikes have not been observed. Therefore, CTC levels early in the course of a new therapeutic regimen for metastatic disease appear to reflect response, whereas lack of reduction of CTC levels may reflect futility of the respective treatment, thus making early CTC levels a short-term “predictive” factor. These data opened a whole new vision of the “personalized medicine” in metastatic breast cancer as no predictive factor—except HR and HER2—have been strongly validated in stage IV breast cancer. In this context, the promise of CTC count was to evaluate the efficacy of any chemotherapy after only one cycle and then to switch non-responding patients to another chemotherapy before the disease progression. However, the clinical benefit of such revolutionary management—compared to the standard radiological evaluation—was unknown. This protocol was the first interventional trial to be designed to demonstrate that CTC-driven management of chemotherapy improves the outcome of metastatic breast cancer patients.

### Design

**Fig. 2:**
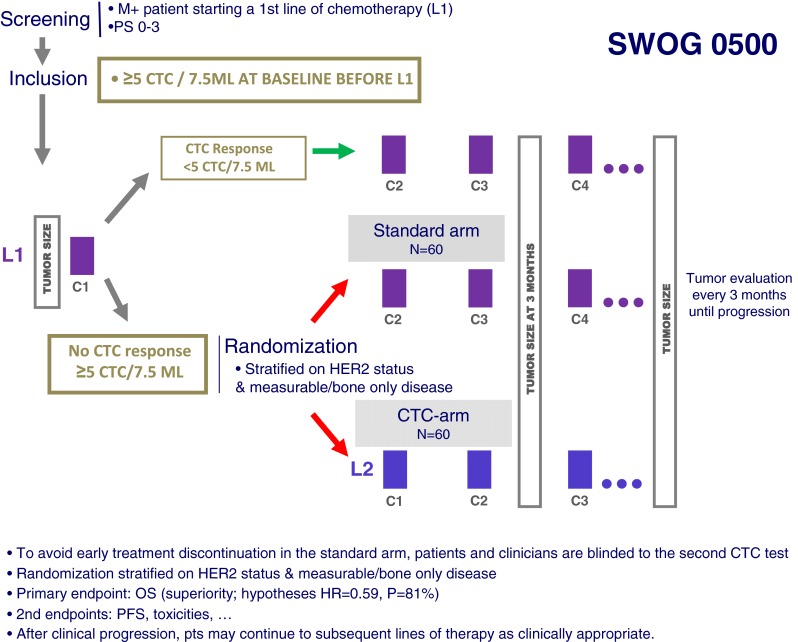
The SWOG 0500 trial design

The SWOG 0500 (NCT00382018) design is shown Fig. 2: in the screening part of the trial, metastatic patients treated by first-line chemotherapy (combined or not with targeted therapy) had their CTC count determined before cycles 1 and 2. Patients with persistently elevated CTC (≥5 CTC/7.5 ml) after one cycle (*i.e.*, around days 21–28) are at higher risk of early cancer progression and were randomized between continuation of the first-line chemotherapy (until classic evidence of clinical or radiological progression) or to early switch to another chemotherapy regimen, before any radiological progression. The “early second line” and further treatment lines were managed by the usual clinical–radiological parameters. Accrual started in 2006 and has been completed in March 2012: about 610 patients were screened and 120 patients randomized (source: www.swogstat.org). The primary endpoint of the trial is to demonstrate an improvement in OS in the CTC-driven arm.

### Funding

The SWOG 0500 trial has been funded by the National Cancer Institute of the USA through the South-West Oncology Group (SWOG), and Veridex supported the study. The promoter is the SWOG.

## CirCe01 (France)

### Rationale

The CirCe01 study is basically another attempt to demonstrate that patients who received a first cycle of chemotherapy and whose CTC count did not drop should be switched off this chemotherapy. Based on the assumption that CTC count is a test to detect chemoresistance, it was supposed that this test will perform better in a population with chemoresistance prevalence, *i.e.*, in the advanced metastatic setting. Another idea is that, to show a clinical improvement in the CTC-managed arm, the CTC test should be repeated more than once in patients. So the early CTC “go/no go” test will be performed not only once, but at the beginning of every new chemotherapy line in patients randomized in the CTC arm (*i.e.*, to evaluate the third, fourth, fifth… lines). In the CTC arm, it has been hypothesized that many patients with chemoresistant tumor will experience repeated early chemotherapy changes (*e.g.*, three chemotherapy regimens tested in 9 weeks), giving support to the discontinuation of inefficient, toxic, and costly chemotherapies and the start of palliative care. It is believed, for this subgroup of patients, that such de-escalating management will benefit both patients and health care systems. For some other patients, it is also expected that they will benefit from the early chemotherapy turnaround and find quicker an efficient therapy among all those tested.

### Design

**Fig. 3:**
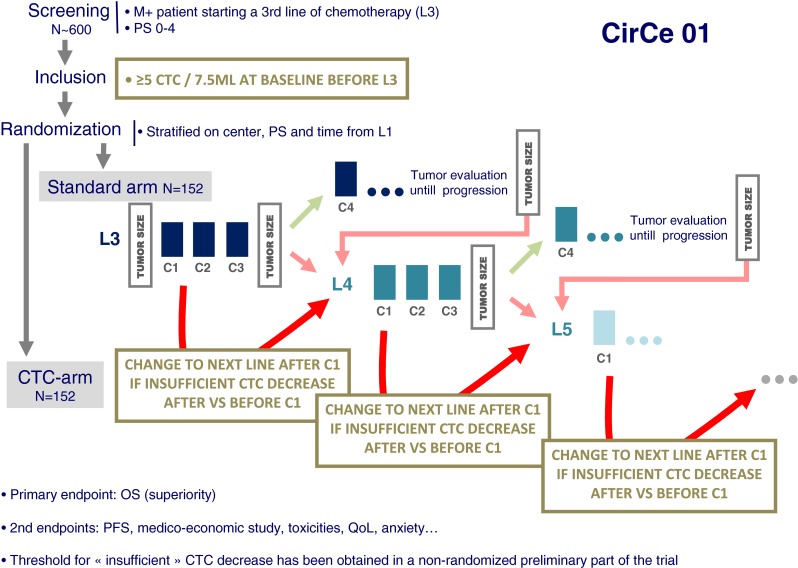
The design of the interventional part of the CirCe01 trial

The first step of the CirCe01 (NCT01349842) trial was conducted from March 2010 to October 2011. This non-interventional step was intended to check the clinical relevance of the ≥5 CTC/7.5 ml threshold in these heavily pretreated patients or to propose alternate decision criteria. The design of the interventional part of the trial, which started in February 2012, is shown in Fig. 3: 304 patients with high CTC count before the start of the third line of chemotherapy will be randomized between the CTC-driven arm and the standard arm. In the CTC-driven arm, CTC counts will be performed after each first cycle of every new chemotherapy drug and will indicate whether or not this regimen is worth to be pursued. Patients with insufficient CTC decrease will be switched off from this chemotherapy line and will be eventually proposed with a further line of treatment, which will be, again, evaluated by early CTC changes (and so on). Patients with a significant CTC decrease before the second chemotherapy cycle will continue their treatment and then managed by standard clinical/radiological tools (the next chemotherapy line being again initially evaluated by CTC, and so on). The medical primary endpoint of the trial is the overall survival, whereas a medico-economic study as a co-primary endpoint. Several secondary endpoints are pre-planned, including progression-free survival, quality of life, anxiety/depression, and comparison with serum markers.

### Funding

The CirCe01 trial has been funded/supported by La Ligue Contre Le Cancer (#20 %), the French ministry of Health (#60 %), and Veridex (#20 %). The promoter is the Institut Curie.

## Treat CTC (Europe)

### Rationale

“Treat CTC” is the first, multicenter international trial assessing CTC detection as a liquid biopsy to test a new treatment strategy in breast cancer that is the use of trastuzumab in HER2 non-amplified disease. Currently, trastuzumab is administered as part of the standard of care only in women with HER2-overexpressing/amplified tumors [17]. However, data from subset analyses of the NASBP B-31 and the NCCTG N9831 trials [18, 19] and results of a single-center phase II study [20] suggest that the benefit from adjuvant trastuzumab may not be confined to HER2-amplified tumors. These interesting findings could be explained by several hypotheses that are not mutually exclusive: (1) lower levels of HER2 expression may be sufficient for benefit from trastuzumab, (2) small undetectable populations of HER2-overexpressing cells may drive benefit from trastuzumab, and (3) trastuzumab may work through eradication of minimal residual disease by immune-related mechanisms. In the Treat CTC trial, we hypothesize that women with HER2-non-amplified, nonmetastatic breast cancer and detectable CTC(s) (irrespective of HER2 overexpression) may benefit from trastuzumab.

### Design

**Fig. 4:**
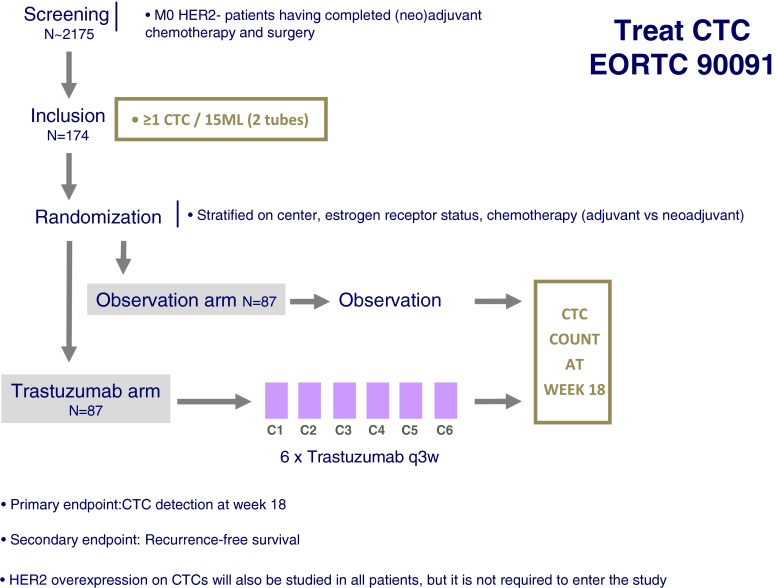
The Treat CTC trial design

The Treat CTC trial (EudraCT 2009-017485-23) flowchart is presented in Fig. 4. This is a randomized phase II trial for patients with HER2-non-amplified primary breast cancer with ≥1CTC/15 ml of blood after completion of (neo-) adjuvant chemotherapy and surgery. Before randomization, a central review on both the HER2 status of the primary tumor and the Cellsearch® CTC images will be performed. Eligible patients will be randomized in 1:1 ratio to either the trastuzumab or the observation arm. Patients randomized to the trastuzumab arm will receive a total of six injections every 3 weeks (loading dose 8 mg/kg IV and 5 cycles at 6 mg/kg every 3 weeks). Patients randomized to observation shall be observed for 18 weeks. The primary endpoint will compare CTC detection rate at week 18 between the two arms, while the secondary endpoint will compare recurrence-free interval.

### Funding

The Treat CTC trial is sponsored by the European Organization for Research and Treatment of Cancer (EORTC) and has been funded/supported by Roche and Veridex. This trial will be conducted under the Breast International Group umbrella.

## DETECT III (Germany)

### Rationale

Patients with a HER2-negative primary tumor can develop HER2-positive CTC during disease progression [21, 22]. The question if these patients benefit from a therapy targeted against HER2 is of particular importance. Case reports have already indicated that initially HER2-negative metastatic breast cancer patients with HER2-positive CTC benefit from trastuzumab-containing therapy regimens [23]. The DETECT III study is a prospective, multicenter, randomized, open-label, two-arm phase III study to compare standard therapy alone *versus* standard therapy plus lapatinib in patients with initially HER2-negative metastatic breast cancer and HER2-positive CTCs (www.detect-studien.de). The small-molecule lapatinib is an inhibitor of tyrosine kinase activity of both HER2 and EGFR [24]. If the DETECT III trial succeeds in proving efficacy as a HER2 treatment in patients with HER2-positive CTCs, a new strategy in treating metastatic breast cancer will be established. The longitudinal analysis of CTC dynamics during follow-up will give new insights into the biology of CTC in metastasizing breast cancer.

### Design

**Fig. 5:**
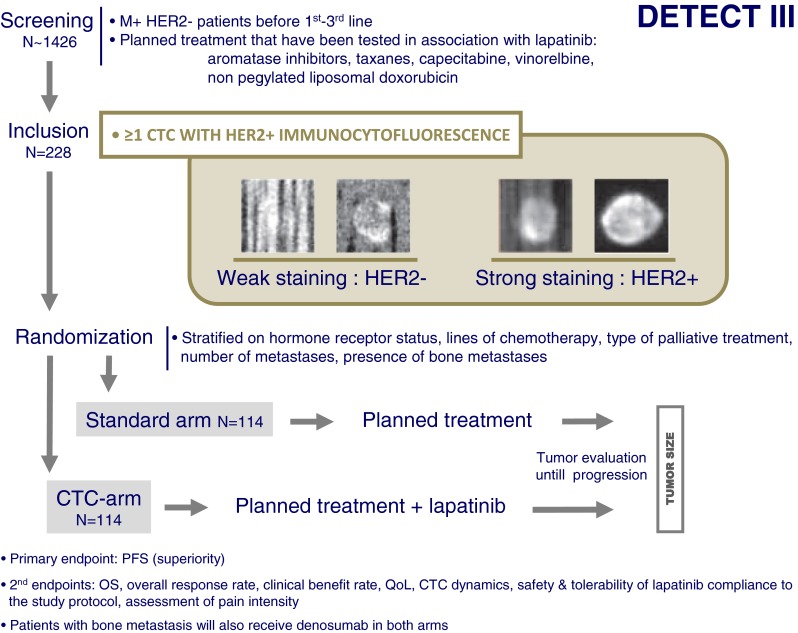
The DETECT III trial design

The DETECT III study (EudraCT 2010-024238-46) started in February 2012. The design of the study is shown in Fig. 5. During the screening phase of the study, 1,426 patients with metastatic breast cancer and with up to three chemotherapy lines for metastatic disease will be tested for HER2-positive CTC. In all patients, the HER2 status of the primary tumor and, if analyzed, of metastatic lesions has to be negative. At least one HER2-positive CTC/7.5 ml blood has to be detected in these patients (immunocytofluorescence). Two hundred twenty-eight patients meeting the inclusion criteria will be randomized between two arms receiving standard therapy or standard therapy plus lapatinib. Standard treatment will consist of chemo- or endocrine therapy that is either approved for the combination with lapatinib or that has been investigated in clinical trials. Patients with bone metastases will be treated with denosumab in both arms. The maximum duration of randomized treatment period is 12 months; the adjacent follow-up period has an estimated maximum duration of 24 months. Primary endpoint of the DETECT III study is progression-free survival; secondary endpoints are overall survival, overall response rate, clinical benefit rate, and the dynamic of CTC.

### Funding

DETECT III is funded by GlaxoSmithKline (82.5 %), Cephalon (15.5 %), and Pierre Fabre Pharma (2 %). The promoter is the Heinrich Heine University Düsseldorf.

### Alternate proposal

**Fig. 6:**
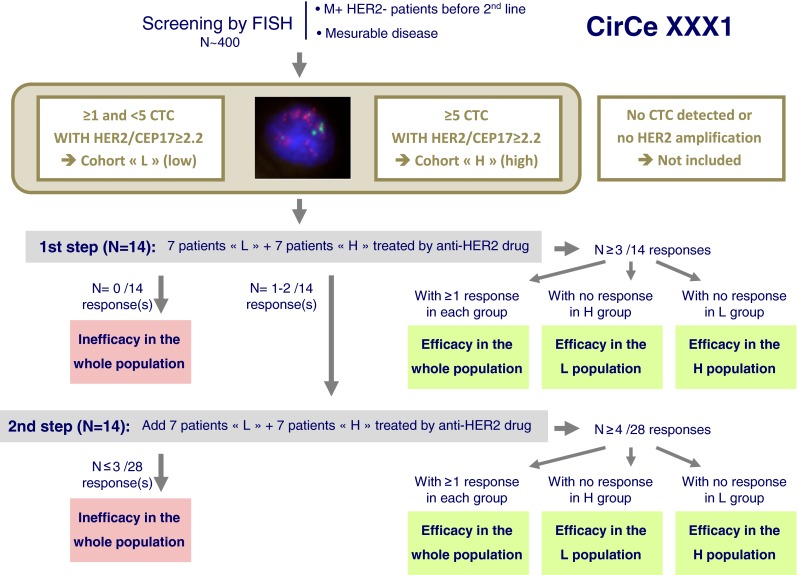
The CirCe XXX1 (provisional name) trial design

Based on the same rationale (HER2 “gain” in CTCs at metastatic stage in HER2-negative primary breast cancers), an interventional phase II study is planned to open in winter 2012 in France. The “CirCe XXX1” study (provisional name) will use the gold standard technique for HER2 amplification assessment that is HER2/CEP17 ratio measurement by FISH. In this single-arm study, patients with HER2-amplified CTC will receive an anti-HER2 drug without combined chemotherapy; response rate will be the study’s main endpoint. It has been anticipated that such strategy might be relevant only in patients with high number of HER2-amplified CTCs; an original adaptive design (Fig. 6) will allow isolating which patient population will benefit (*i.e.*, the “granularity” of the screening procedure).

## Conclusion

Beyond the future of CTC analysis as a biological tool for understanding of the metastatic process, the CTC count is clinically a very promising tool. This new dynamic quantitative and qualitative test deserves an appropriate scientific development in order to be efficient and to further improve the life expectancy and/or the quality of life of breast cancer patients. Each of the interventional trials presented above has been designed using the CellSearch® system, as this system has the largest background of non-interventional studies published in the past years. This background allowed the accumulation of clinical data and hypotheses, of which statistical hypotheses used for such large studies. Interestingly, these studies are not part of pre-planned development program and are addressing different but complementary aspects of the CTC clinical utility in different parts of the world. For example, a similar test result with stable intrinsic statistical properties (lack of early CTC changes during chemotherapy) can be used to reach different objectives: in SWOG0500, the underlying concept is to intensify the frontline treatment for the few metastatic patients who are not responding. In contrast, in CirCe01, the underlying concept is to objectively justify the early discontinuation of useless chemotherapies for the vast majority of patients who are in a very palliative setting. It is also expected that the global accuracy of the CTC changes, used as a test for early chemoresistance detection, will vary between these two trials, as chemoresistance rates are different between the first and the latest chemotherapy lines.

Another interesting aspect is the different trials proposed to assess the relevance of CTC detection and characterization as a “liquid biopsy” to test new treatment strategy using anti-HER2 drugs: the DETECT III trial uses a classical randomized phase III trial design and will answer in a large population whether or not lapatinib should be added in a HER2− breast cancer patients population that has been predefined before the start of the study: metastatic patients with any HER2+ CTC by immunocytofluorescence. A very different approach is lead by the Treat CTC trial, which combines the prognostic information of CTC in the adjuvant setting (cM0(i+) patients[25]) and the promise of adjuvant trastuzumab given to HER2-negative patients in past studies. Interestingly, the adjuvant setting is characterized by low CTC detection rates and by long follow-up, explaining why this trial is the only one not using survival as first endpoint. Also, the HER2 status of CTC is not used as inclusion criteria but will be registered, leading to possible subgroups analysis of trastuzumab efficacy in this high-risk population.

Finally, these trials represent the first attempts to demonstrate that CTC testing improve the clinical outcome of metastatic breast cancer. Each of them proposes a different original design which may guide the setup of further studies based on dynamic blood markers.
